# Intussusception of the cecum due to the acute appendicitis: A case report

**DOI:** 10.1016/j.ijscr.2022.107727

**Published:** 2022-10-12

**Authors:** Naoya Kimura, Masatsugu Hiraki, Hirofumi Sato, Hiroki Koga, Daisuke Mori, Toshiya Tanaka, Kenji Kitahara

**Affiliations:** a Department of Surgery, Saga Medical Center Koseikan, 400 Nakabaru, Kasemachi, Saga City, Saga 840-8571, Japan; b Department of Pathology, Saga Medical Center Koseikan, 400 Nakabaru, Kasemachi, Saga City, Saga 840-8571, Japan

**Keywords:** Intussusception, Appendicitis, Bowel obstruction

## Abstract

**Introduction and importance:**

Intussusception of the cecum due to acute appendicitis is rare condition.

**Presentation of case:**

A 17-year-old male patient presented to our hospital with a chief complaint of right lower abdominal pain, which had lasted for two days. Computed tomography (CT) revealed a “target sign” from the cecum to the ascending colon, leading to a diagnosis of cecocolic intussusception. Colonoscopy revealed an erythematous, edematous, and internally distorted cecum in the ascending colon, which was difficult to repair with air insufflation. Laparoscopic surgery was performed to remove the bowel obstruction. Repositioning of the invaginated cecum was difficult due to the presence of a hard and edematous colic wall. Therefore, laparoscopic ileocecal resection was performed to release the obstruction. The pathological diagnosis was appendicitis and abscess within the cecum wall, with no malignant findings.

**Discussion:**

In our case, intussusception was considered to have caused thickening of the intestinal wall of the cecum due to inflammation of the appendix, and the thickened area became the leading point.

**Conclusion:**

Considering that malignancy is a frequent leading point in adult patients with intussusception, a preoperative endoscopic examination is important for minimizing bowel resection.

## Highlights

• Most cases of intussusception outside of childhood are caused by tumors, but inflammation of the appendix or cecum can be a cause.
• If malignancy is not the cause of intussusception, bowel resection can be minimized.
• In order to determine the appropriate extent of resection, an accurate preoperative diagnosis is necessary, keeping in mind the possibility that appendicitis or inflammatory changes in the cecum may be the leading point.

## 1 Introduction

Intussusception is a condition that a portion of the intestine invaginates within the distal segment [1]. In young children, up to 90 % of cases of intussusception are reported to be idiopathic. On the other hand, intussusception in adults is rare, accounting for only 5 % of all cases of intussusception in all ages; in most cases tumors are reported to be the leading point [3], [4]. Therefore, intussusception caused by inflammatory disease is a rare condition with only a very small number of cases reported in the relevant literature [5], [6], [7], [8], [9], [10], [11].

We herein report a case of cecocolic intussusception caused by inflammation of the cecum due to appendicitis. This work has been reported in line with the SCARE criteria [12].

## 2 Presentation of case

**Fig. 1:**
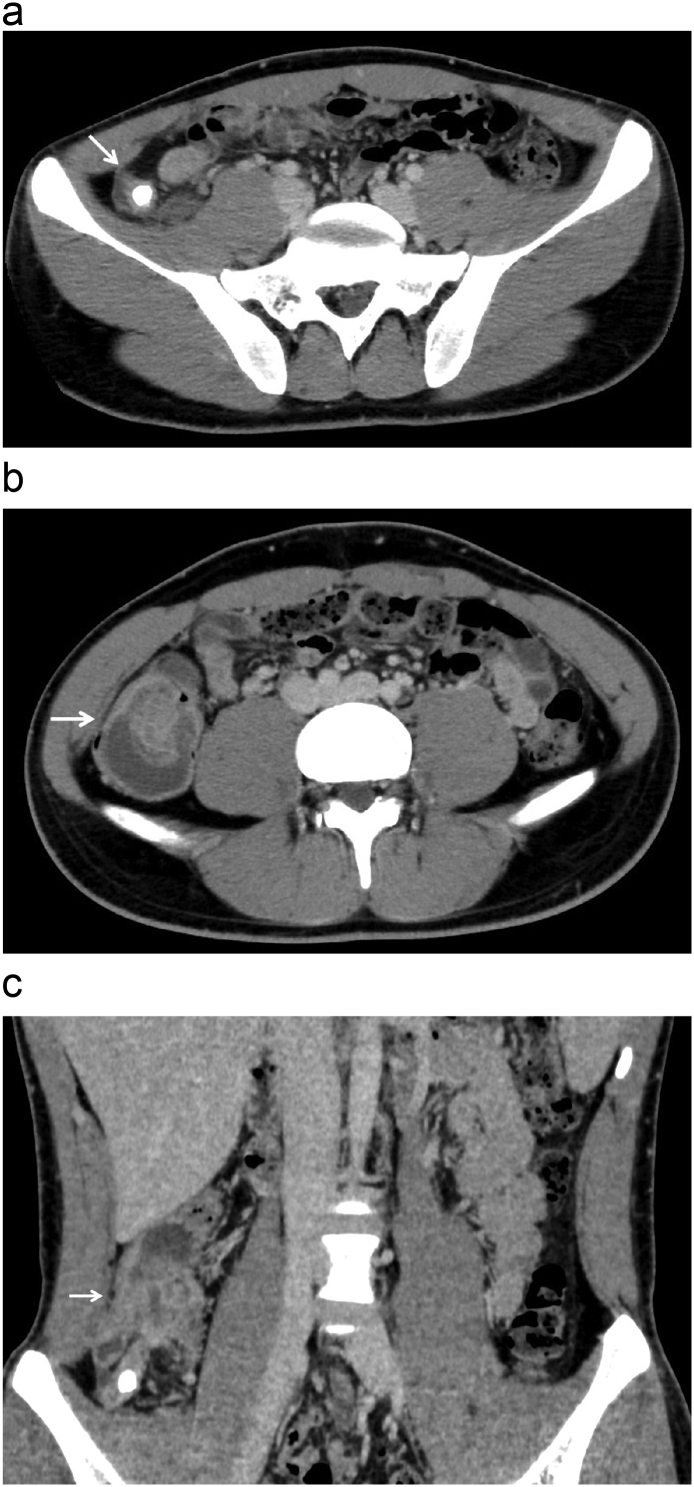
Computed tomography (CT) showed the swollen appendix and appendicolith in the middle of the appendix (a: axial plane) (white arrow). The target sign indicated that the cecum was intussuscepted to the ascending colon (b: axial plane) (white arrow). The colonic wall of the cecum and ascending colon showed edematous thickening (c: sagittal plane) (white arrow).

**Fig. 2:**
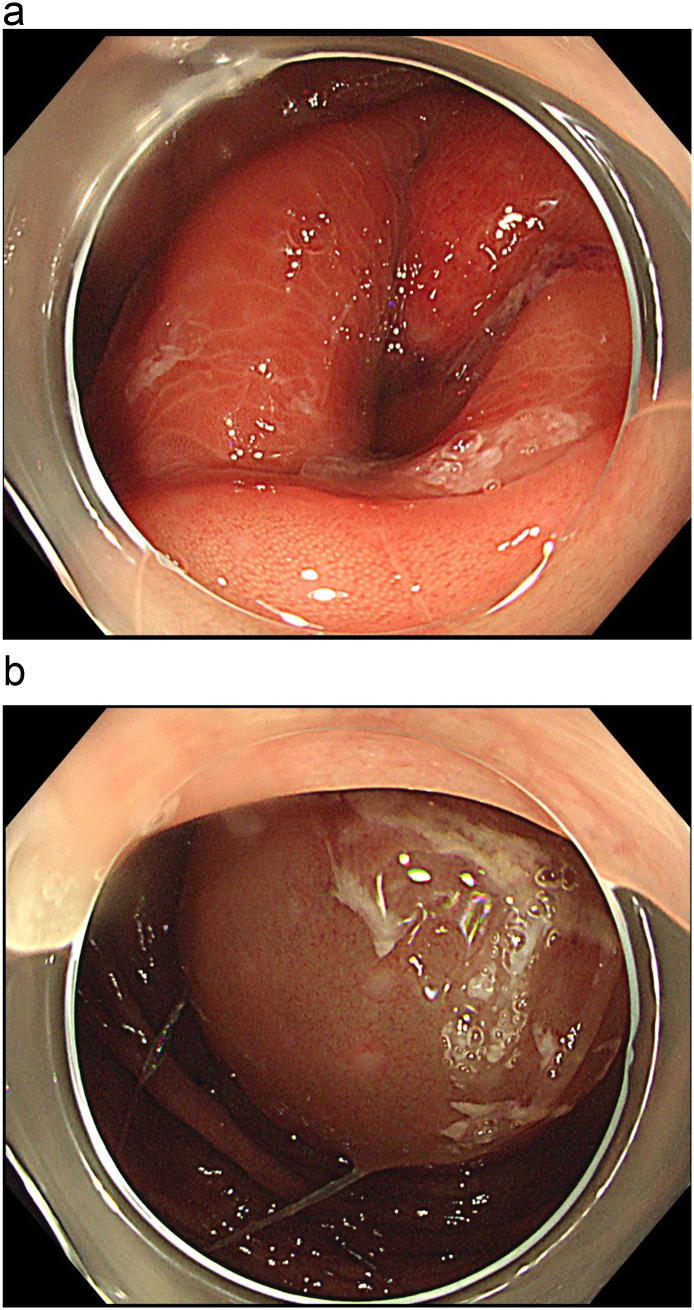
Colonoscopy demonstrated edematous changes of the ileocecal valve (a). The wall of the cecum and ascending colon also showed edematous change (b).

**Fig. 3:**
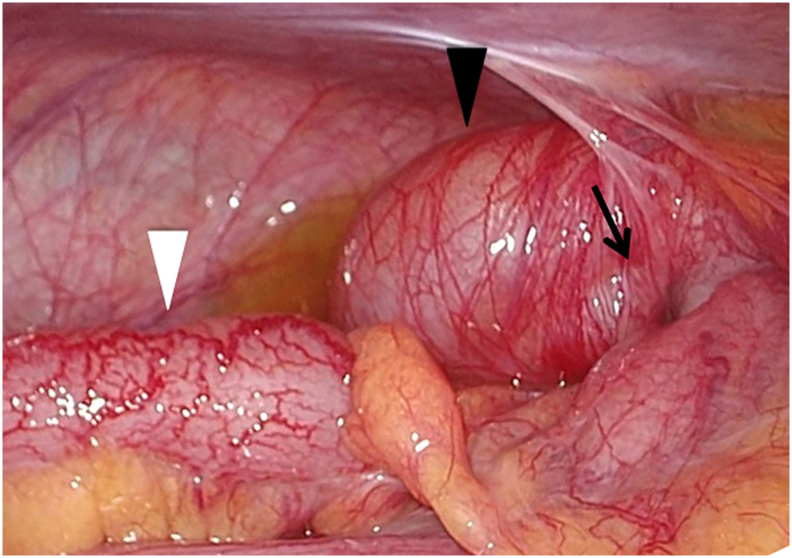
Laparoscopic observation of the swollen appendix (white arrowhead). The thickened cecum with edema (arrowhead) was intussuscepted into the wall of the ascending colon (black arrow).

The patient was a 17-year-old boy who presented to hospital with the right lower abdominal pain that had persisted for two days. The patient had no relevant history and was not using medication. His body temperature was 37.3 °C. Physical examination demonstrated tenderness around McBurney's point. Muscular defense and Blumberg's sign were absent. Laboratory studies showed elevated inflammatory findings (white blood cells: 12.0 × 10^3^/μl, C-reactive protein: 1.6 mg/dl). Computed tomography (CT) showed a swollen appendix and appendicolith in the middle of the appendix (Fig. 1a). The “target sign” indicated that the cecum was intussuscepted to the ascending colon, and the colonic wall of the cecum and the ascending colon also showed edematous thickening (Fig. 1b, c). Colonoscopy demonstrated edematous changes of the ileocecal valve, cecum, and ascending colon (Fig. 2a, b). CT and colonoscopy revealed no signs of tumor. A diagnosis of acute appendicitis, and intussusception of the cecum were made. We were concerned about bowel obstruction due to intussusception and treatment for appendicitis was needed. Therefore, a laparoscopic operation was performed. The operative findings revealed thickening of the colonic wall of the cecum with edema, and the cecum was intussuscepted to the ascending colon. Repositioning of the invaginated cecum was therefore attempted in order to obtain better understanding of the proper anatomical position. However, it was difficult due to the hard and edematous colic wall. Therefore, laparoscopic ileocecal resection was performed to release the obstruction (Fig. 3).

**Fig. 4:**
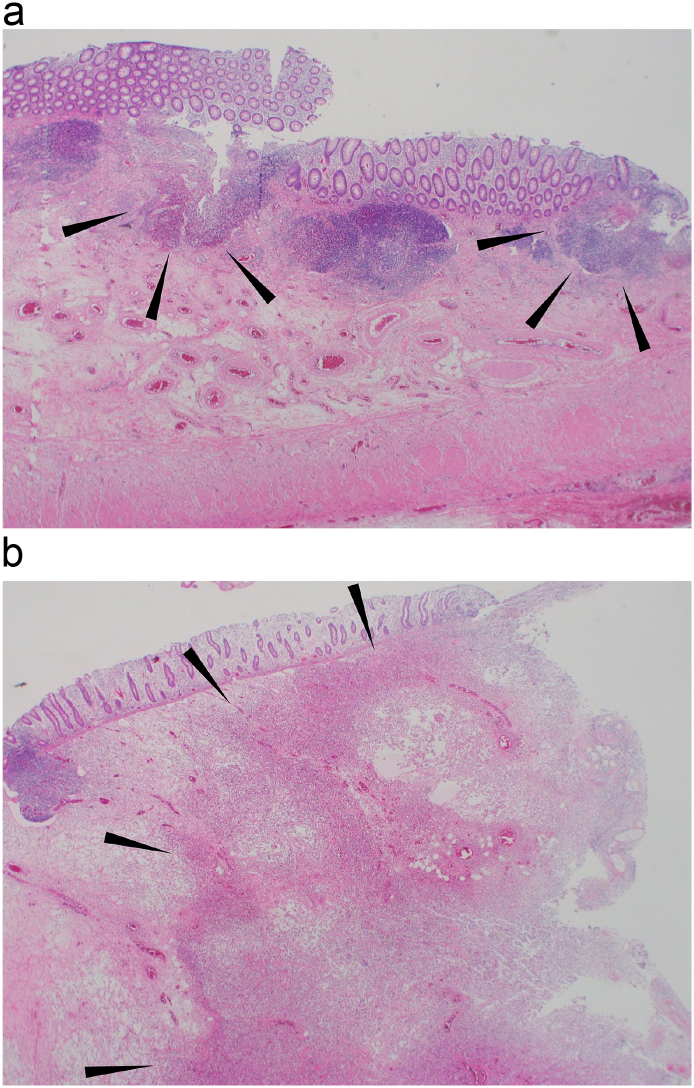
Pathological examinations showed edema and the infiltration of inflammatory cells at the appendix (a: HE, ×40) (black arrowhead). Formation of the abscess and bacterial accumulation was observed inside the intestinal wall of the ileocecum (b: HE, ×40) (black arrowhead).

A pathological examination showed edema and infiltration of inflammatory cells at the appendix (Fig. 4a). Formation of an abscess and bacterial accumulation inside of the intestinal wall of the ileocecum were seen (Fig. 4b). Fibrotic change was seen at the serosa and the mesentery of the appendix. No malignant lesion was detected. The patient was discharged from hospital on postoperative day 6.

## 3 Discussion

In adults, intussusception is a relatively rare condition in comparison to children, accounting for only 5 % of all cases of intestinal intussusception [3]. While >90 % of pediatric cases are idiopathic, >90 % of cases in adults occur with organic disease (e.g., benign or malignant tumors) as a leading point, and malignant tumors account for approximately 60 % of cases of colorectal intussusception [4], [13], [14]. Intussusception can be broadly classified according to location (small intestine, ileum, colon) and cause (benign tumor, malignancy, idiopathic), and intussusception without a leading point has been reported to be a transient, incidental finding in adults with celiac disease or Crohn's disease [15].

While intussusception of the appendix itself caused by appendicitis has been reported [16], a search of the PubMed database using the keywords “intussusception” and “appendicitis” and “ileocolic” or “cecocolic” revealed only seven reports of cecocolic intussusception caused by appendicitis as a secondary disease from April 1955 to April 2022 (excluding conference proceedings and our case) [5], [6], [7], [8], [9], [10], [11]. Table 1 shows the eight reported cases of cecocolic intussusception caused by appendicitis, including the present case.

**Table 1:** Patients' clinical characteristics.

No.	Author	Year	Age	Sex	Symptom	Preoperative examination	Treatment	Pathological diagnosis
1	Kang J	2014	73	Female	Right lower abdominal pain	CT	Laparoscopic right hemicolectomy	Appendicitis
2	Nelson MJ	2014	6	Male	Right lower abdominal pain	Abdominal ultrasound, CT	Conservative treatment	Appendicitis
3	Kee HM	2015	3	Male	Right lower abdominal pain	Abdominal ultrasound	Lparoscopic appendectomy	Appendicitis
4	Marjon L	2018	1	Male	Fussiness, vomiting, and red gelatinous stools	Abdominal ultrasound	Laoaroscopic appendectomy	Appendicitis
5	Ravikanth R	2021	4	Male	Right abdominal pain	Abdominal ultrasound	Laproscopic appenditectomy	Appendicitis
6	Ebrahimi N	2021	22	Male	Right abdominal pain and fever	CT	Laparoscopic right hemicolectomy	Appendicitis
7	Furlong SA	2022	2	Female	Abdominal pain, fussiness, anorexia, and lethargy	Abdominal ultrasound	Laparoscopic appendectomy	Appendicitis
8	Our case	2022	17	Male	Right lower abdominal pain, rebound tenderness and fever	Computed, colonoscopy	Laparoscopic ileocecal resection	Appendicitis

*CT* computed tomography.

Six patients (75 %) were children (<18 years of age), one patient (12.5 %) was in his 20s, and one patient (12.5 %) was in his 70s. The majority of cases involved young patients. Therefore, in childhood, it may be related to the fixation and mobility of the cecum and ascending colon to the retroperitoneum. In addition, in adults, it may be due to a lack or loosening of fusion of the cecum to the retroperitoneum.

Among the eight reported cases of cecocolic intussusception caused by appendicitis, the most common symptom was abdominal pain associated with intestinal obstruction (87.5 %), followed by nausea and vomiting (12.5 %), and fever (25.0 %). Bloody and gelatinous stools were also observed in pediatric patients. The most common symptoms of intussusception are reported to be due to bowel obstruction: cramping abdominal pain (71 %), nausea and vomiting (68 %), abdominal distention (45 %), and tenderness (60 %); however, these symptoms are often absent in adults [17], [18]. Our case presented right lower abdominal pain due to appendicitis rather than symptoms of intestinal obstruction due to the intussusception. Taken together, this disease must be considered to present with physical findings of appendicitis and/or bowel obstruction due to intussusception.

Abdominal ultrasonography and CT are commonly used for the diagnosis of intussusception. Its characteristic findings include the “target sign” and “doughnut sign” in transverse sections, and the “hay fork sign” and “pseudo kidney sign” in longitudinal sections [19]. Abdominal CT is also helpful for detecting the presence of leading points, such as a tumorous lesion and/or intestinal calculus [20], [21]. In addition to ultrasonography and CT, colonoscopy may be useful for the definite diagnosis because benign or malignant tumors can be a leading point in intussusception in adults. Kang et al. [5] also experienced similar cases and reported that intussusception of the cecum into the ascending colon and preoperative colonoscopy demonstrated the absence of neoplastic lesions of the intestinal tract. They performed right hemicolectomy because they could not rule out the possibility of a malignant tumor at the leading point that had not been identified by extra-intestinal observation. In our case, we were able to limit the extent of resection because we were able to deny the presence of a tumor by colonoscopy. Considering that most adult patients with intussusception have a malignant tumor as a leading point, it is strongly recommended that malignant findings be ruled out preoperatively. Regarding the diagnosis of eight cases of cecocolic intussusception caused by appendicitis, CT was performed in only half of the cases (50 %), and echocardiography led to the diagnosis of intestinal polyposis in 5 cases (67.5 %). This might be due to the high incidence of this disease in pediatric patients.

In our case, the pathological findings revealed submucosal edema and inflammatory cell infiltration in the appendix, which led to a diagnosis of acute appendicitis. In previous reported cases appendicitis was also diagnosed pathologically (Table 1). There were no findings of malignancy. Pyogenic inflammation was present around the wall of the appendix, from the root of the appendix to the cecum. Based on these results, it was considered that the mechanism of the disease was thickening of the intestinal wall of the cecum triggered by pyogenic appendicitis as the leading point.

In eight cases of cecocolic intussusception caused by appendicitis, laparoscopic surgery was performed in 7 cases (87.5 %); only one pediatric case (12.5 %) was relieved by conservative treatment. Among the 7 patients who were treated by surgery. Laparoscopic appendectomy and bowel resection were performed in 4 and 3 patients, respectively. In cases in which intussusception and bowel obstruction are difficult to remove, it is necessary to consider bowel resection.

## 4 Conclusion

We experienced a case of cecocolic intussusception caused by acute appendicitis. Surgical treatment was safely performed based on an accurate and appropriate preoperative diagnosis.

## Funding

None.

## Ethical approval

Not applicable in this case report as it is not a research study.

## Consent

Written informed consent was obtained from the patient for publication of this case report and accompanying images. A copy of the written consent is available for review by the Editor-in-Chief of this journal on request.

## Provenance and peer review

Not commissioned, externally peer-reviewed.

## Author contribution

All the authors contributed to diagnose and treat the patient. Naoya Kimura and Masatsugu Hiraki contributed in drafting the manuscript. Toshiya Tanaka and Kenji Kitahara supervised and made the final approval of the manuscript. All authors read and approved the final manuscript.

## Research registration

None.

## Guarantor

Toshiya Tanaka, Department of Surgery, Saga Medical Center Koseikan.

## Declaration of competing interest

The authors declare no conflicts of interest in association with the present study.
